# Items

**Published:** 1883-04

**Authors:** 


### Items.

METEOROLOGICAL DATA.—For the month of Feb., 1883. E. A. Beals, Observer, U. S. Signal Station, Atlanta, Ga.

| | | |
|---|---|---|
| BAROMETER | —Mean | 30.313 |
| | Highest | 30.664 on 27th |
| | Lowest | 30.081 on 7th. |
| | Range | .583 |
| THERMOMETER | —Mean | 49.8 |
| | Highest | 74.5 on 16th. |
| | Lowest | 28.0 on 27th. |
| | Range | 46.5 |
| | Greatest Daily Range | 29.4 on 4th. |
| | Least Daily Range | 3.0 on 10th. |
| Humidity | —Mean | 76.9 |
| Dew-point | —Mean | 41.6 |
| Rainfall | —Total | 3.22 inches. |
| | Greatest Daily | .58 on 15th. |
| | Total Number of Days | 18 |
| Wind | —Prevailing Direction | N. W. |
| | Maximum Velocity | N. W., 30 miles per hour. |

---

THE new year witnessed great improvement in the medical periodical literature of the United States. Monthlies were changed into weeklies, many weeklies were enlarged, besides giving other evidences of a vigorous existence. These are encouraging indications, and show not only enterprise and activity upon the part of publishers, but appreciation upon the part of the medical public.

---

A JURY recently returned a verdict against New York city for $25,000 in favor of a man who was injured by running his wagon into a hole in the street. At the trial Drs. Janeway and Sayre testified that the resulting trouble was Potts' disease, while Drs. Hammond, Hamilton, Clymer and Stimson testified that he was suffering from sclerosis of the cord, due to cold and exposure.

When doctors disagree a petit jury decides.

---

HEAR what the *Medical and Surgical Reporter* saith:

"We fear and hope that specialism, carried to the extent it now is, will soon prove one of the schisms with which our profession has been, from time immemorial, afflicted, and will live its day, to give place again to the two regular and legitimate divisions, viz., medicine and surgery."

---

NEW YORK CITY has appropriated $50,000 for building a hospital to be used exclusively for the treatment of scarlet fever and diphtheria.

---

THE next session of the International Medical Congress will be held at Copenhagen, in August, 1884.

---

FAILURES, rightly interpreted, strengthen the character no less than successes temperately enjoyed.

---

"THOUGH learning guides us healing herbs to pick,
'Tis wisdom only makes them cure the sick."

---

REVERSES are not always failures.

